# Instability of the Middle Cerebral Artery Blood Flow in Response to CO_2_

**DOI:** 10.1371/journal.pone.0070751

**Published:** 2013-07-30

**Authors:** Rosemary E. Regan, James Duffin, Joseph A. Fisher

**Affiliations:** 1 Department of Physiology, University of Toronto, Toronto, Ontario, Canada; 2 Department of Anaesthesia, University of Toronto, and University Health Network, Toronto, Ontario, Canada; Kaohsiung Chang Gung Memorial Hospital, Taiwan

## Abstract

**Background:**

The middle cerebral artery supplies long end-artery branches to perfuse the deep white matter and shorter peripheral branches to perfuse cortical and subcortical tissues. A generalized vasodilatory stimulus such as carbon dioxide not only results in an increase in flow to these various tissue beds but also redistribution among them. We employed a fast step increase in carbon dioxide to detect the dynamics of the cerebral blood flow response.

**Methodology/Principal Findings:**

The study was approved by the Research Ethics Board of the University Health Network at the University of Toronto. We used transcranial ultrasound to measure the time course of middle cerebral artery blood flow velocity in 28 healthy adults. Normoxic, isoxic step increases in arterial carbon dioxide tension of 10 mmHg from both hypocapnic and normocapnic baselines were produced using a new prospective targeting system that enabled a more rapid step change than has been previously achievable. In most of the 28 subjects the responses at both carbon dioxide ranges were characterised by more complex responses than a single exponential rise. Most responses were characterised by a fast initial response which then declined rapidly to a nadir, followed by a slower secondary response, with some showing oscillations before stabilising.

**Conclusions/Significance:**

A rapid step increase in carbon dioxide tension is capable of inducing instability in the cerebral blood flow control system. These dynamic aspects of the cerebral blood flow responses to rapid changes in carbon dioxide must be taken into account when using transcranial blood flow velocity in a single artery segment to measure cerebrovascular reactivity.

## Introduction

The anatomy of the cerebral vasculature is complicated, involving groups of long end-arteries, such as those supplying the deep white matter, and well collateralized vascular networks, such as those supplying the grey matter and subcortical white matter [1]. These vasculatures differ as well in their hemodynamic response to a global carbon dioxide (CO_2_) stimulus with respect to the magnitude, time course, and speed of response. Studies using Magnetic Resonance Imaging (MRI) and Positron Emission Tomography (PET) have shown that the cerebrovascular reactivity of white matter is delayed and about one third of the amplitude of grey matter [2]–[6].

A short distance after its branching from the internal carotid artery, the middle cerebral artery (MCA) gives rise to the lenticulostriate arteries, which are long penetrating end-arteries supplying the deep white matter. More distally, the middle cerebral artery MCA provides perfusion to the cortex and subcoritcal tissues of the parietal and temporal lobes. Like other major brain vessels, the MCA has a higher flow resistance than comparable feeding arteries to other organs [7], and this high blood flow resistance limits its capacity to match any decreased downstream resistance with increased flow. Consequently a generalized vasodilatory stimulus is likely to result in a redistribution of blood flow between co-dependent vascular territories on the basis of their relative reductions in blood flow resistances [8]–[10]. Any differences in the temporal pattern of reduction in resistance between the territories will affect the time course and pattern of blood redistribution and flows through the larger arteries such as the MCA.

A commonly used approach to study such a complicated physiological control system is to apply a standardised input disturbance such as a step function [11]. Previous experiments employing a step function input [12] reported finding a single exponential rise to a final value with an estimated mean time constant of 45 s. However, these authors also noted that the response to a hypercapnic step may be more complicated than a single exponential curve. Indeed, they reported that in 4 of their 6 subjects multiple response components could be identified. We note that the time resolution in the reported data was low due to the low sampling frequency and extensive time averaging in the data analysis. Our aim therefore was to revisit this issue by studying the dynamics of the MCA flow velocity responses to an isoxic step change in arterial CO_2_ tension using a prospective targeting control system capable of controlling arterial CO_2_ tension and end-tidal oxygen (O_2_) tension. We found responses that have not been noted in previous studies, with complex patterns including oscillations that indicate instability.

## Methods

### Subjects and Ethical Approval

These studies conformed to the standards set by the latest revision of the Declaration of Helsinki. After approval from the Research Ethics Board at the Toronto General Hospital (University Health Network) and written informed consent, 28 (18 M) healthy normotensive, non-smoking subjects of mean (SD) age 26 (4) years participated in this study. Subjects were not taking any medication other than oral contraceptives, and had no history or symptoms of cardiovascular, cerebrovascular, or respiratory diseases. All subjects abstained from caffeinated or alcoholic beverages and vigorous exercise for at least 12 hours before the study.

When designing this study, we wished to control for the potential and unknown complication of hormonal impact on vasodilatory reactivity. Premenopausal women have a heightened vasodilatory response [13], [14], which appears to be related to the fluctuation of progesterone and oestrogen [15]. Both Kastrup et al. [16], and Diomedi et al. [17] found evidence for an effect of estrogens. In addition, the small numbers of studies comparing male and female CBF responses to hypercapnia have found evidence for differences. Therefore, all data from female subjects was collected during the first 5 days of their menstrual cycle to minimize the effects of oestrogen and progesterone and eliminate menstrual cycle phase as a variable.

### Apparatus

Middle cerebral artery blood velocity (MCAv) was measured using bilateral trans-cranial Doppler (ST3 Transcranial Doppler, Spencer Technologies; Seattle, USA) at 2 MHz and sampled at 125 Hz. The Power Motion (M-mode) Transcrandial Doppler (PM-TCD) had the advantage of displaying signal intensity in an M-mode format (colour coded direction, simultaneously detectable over 6 cm of intracranial space). In several subjects both the M1 and M2 could be simultaneously displayed while selecting depths as described in [18]. Alexandrov et al. [19] sets the depth range for the M1 to be 40–65 mm, and approximately 80% of patients have their M2 located in the depth range of 30–40 mm [18]. To improve upon this percentage, we selected a more stringent range >45 mm, which is a frequently used standard for identification of the M1 [18].

Subjects were fitted with a face mask, which was connected to the breathing circuit via a mass flow sensor (AWM720P1 Airflow, Honeywell; Freeport, Illinois, USA) to monitor ventilation (Ve). Mean arterial pressure (MAP) and heart rate (HR) were determined by finger plethysmography (Nexfin, BMEYE; Amsterdam, The Netherlands) sampled at 200 Hz. Tidal gas was continuously sampled and analyzed for CO_2_ and O_2_ tensions (RespirAct™, Thornhill Research Inc., Toronto, Canada) and recorded at 20 Hz. Each of these instruments saved a digital record for later analysis.

### Protocol

Subjects were seated upright in a small room facing away from the monitors so that measurement conditions were standardised thereby precluding differences in arousal. No other individuals were allowed in the room at the time of study other then the investigator and subject. Cell phones etc. were turned off during the study. Visual stimuli were avoided and subjects were not cued about the stimuli they were receiving.

We measured the dynamic responses of MCAv, MAP and HR to step increases in CO_2_ tension from 5 mmHg below resting to 5 mmHg above resting (Hypo-Hyper) and from resting to10 mmHg above resting (Normo-Hyper) while maintaining O_2_ tensions at resting levels. Control of end-tidal partial pressures of CO_2_ (PetCO_2_) and O_2_ (PetO_2_) was achieved via prospective targeting, using a sequential gas delivery circuit and a computer driven gas blender (RespirAct™; Thornhill Research Inc, Toronto, Canada) as described by Slessarev et al. [20]. This gas targeting method has been shown to accurately reflect the actual independent variable, the partial pressure of carbon dioxide in arterial blood (PaCO_2_) [21], [22], allowing breath-by-breath resolution of the independent stimulus.

Subjects were seated in a comfortable chair and asked to breathe in time to a metronome at a frequency of 15 b/min, and empty the inspiratory bag of the breathing circuit on each breath, thereby allowing the RespirAct™ complete control of alveolar ventilation [20]. Flow from the computer driven gas blender was adjusted to suit each subject’s resting ventilation and then maintained constant for the experiment. Subjects were maintained at their starting conditions (normocapnia or hypocapnia) for 5 minutes and then their PetCO_2_ was targeted to a step increase of 10 mmHg (achieved within 1–2 breaths) for 10 minutes, after which it was returned to their starting end-tidal tensions for 5 minutes. The PetO_2_ was maintained at resting levels for the entire sequence. Twenty-eight subjects were tested with the two sequences, the order of which was randomised, and at least 20 minutes of rest occurred between the two sequences.

### Data Analysis

For each test, beat-by-beat values of MAP and HR and 4-s averages of MCAv were recorded. Then these measures were time aligned with breath-by-breath Ve, PetCO_2_ and PetO_2_ measures, and plotted vs. time. The pre-step values were calculated as the mean of the starting condition. The larger of the left and right MCAv step responses were then examined and classified as either a single phase response or a response divisible into two phases; initial and secondary, based on the existence of a clear nadir between them. The time delay from the start of the response to the nadir was noted.

The variability of PetCO_2_ and PetO_2_ during the control and hypercapnic periods was measured as the standard deviation of their breath-by-breath values. An exponential rise was fitted to the initial responses in order to determine the rise time constant. This curve fitting was aided by a specially-written program (National Instruments Inc, LabVIEW) that utilised the Levenberg-Marquard algorithm.

## Results

All 28 subjects completed both the Normo-Hyper and the Hypo-Hyper experiments. In all tests the step changes in PetCO_2_ were completed within 2 breaths, i.e. the second breath of the hypercapnic period was at the final value. Blood pressure was unchanged throughout the testing period for the Hypo-Hyper tests. During the Normo-Hyper tests blood pressure rose slowly over the course of the increased PetCO_2_ for half of the subjects by a mean (SD) of 9.9 (2.7) mmHg.

For the Normo-Hyper tests the measured mean (SD) increase in PetCO_2_ was 9.4 (1) mmHg and the mean (SD) change in PetO_2_ from control was 1.3 (1.6) mmHg. During the pre-step period the mean standard deviation of the breath-by-breath variation in PetCO_2_ was 0.2 mmHg and in PetO_2_ was 0.1 mmHg. During the hypercapnic period the mean standard deviation of the breath-by-breath variation in Pet_CO2_ was 0.6 mmHg and in PetO_2_ was 0.5 mmHg.

For the Hypo-Hyper tests the mean (SD) increase in PetCO_2_ was 8.5 (0.9) mmHg and the mean (SD) change in PetO_2_ from control was 1.3 (2.2) mmHg. During the pre-step period the mean standard deviation of the breath-by-breath variation in Pet_CO2_ was 2.4 mmHg and in PetO_2_ was 1.2 mmHg. During the hypercapnic period the mean standard deviation of the breath-by-breath variation in Pet_CO2_ was 1.6 mmHg and in PetO_2_ was 1.0 mmHg.

**Figure 1:**
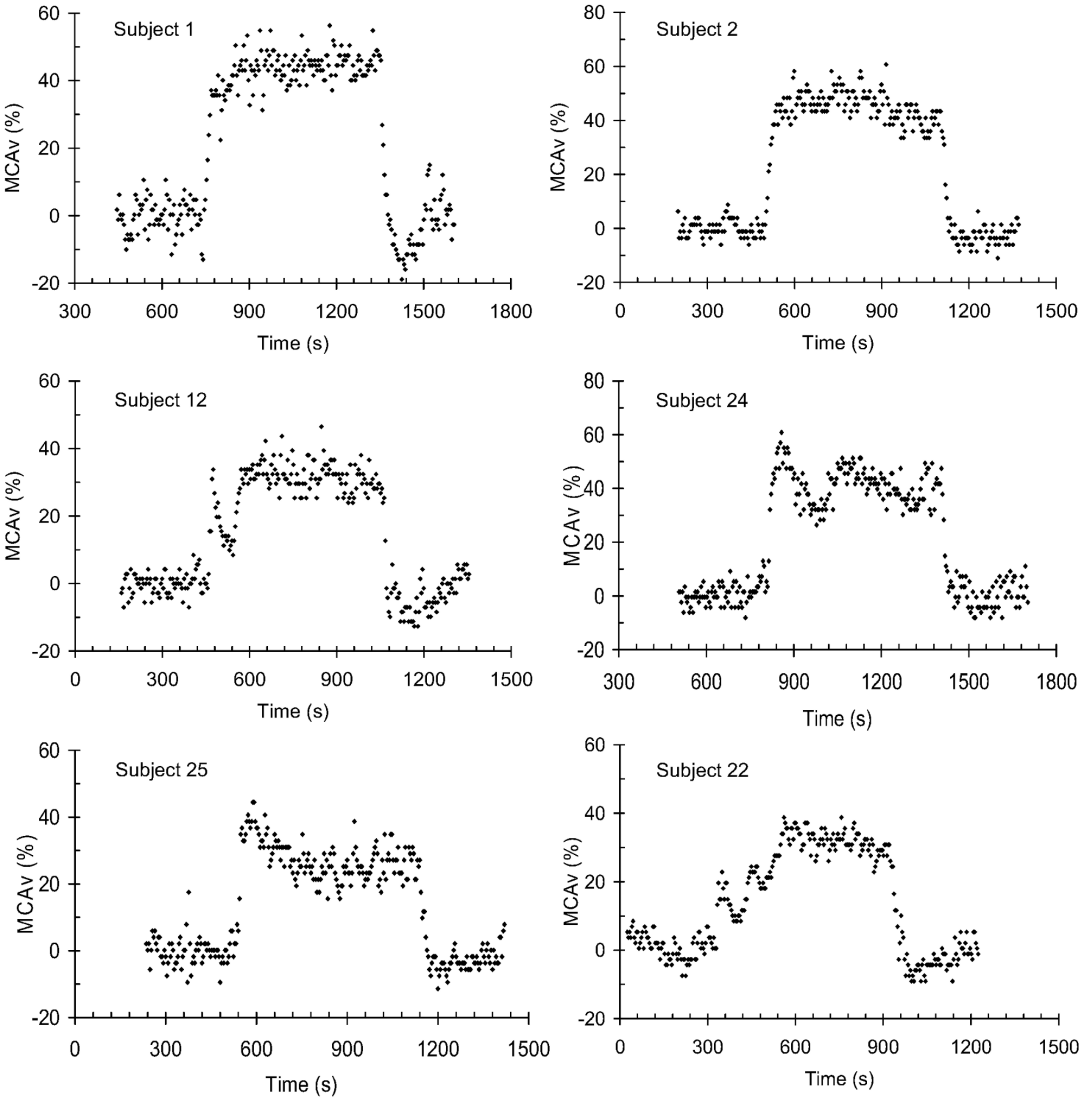
Examples of the MCAv responses to isoxic step increases in PetCO_2_ of 10 mmHg. The points show the 4-s averages of the larger of the left and right MCAv recordings. Plots showing all recorded variables are shown in supplementary information. Subject 1, a single phase response showing a rise to a plateau (Normo-Hyper test); Subject 2, a single phase response showing a rise to a peak followed by a decline (Normo-Hyper test); Subject 12, a two phase response showing a primary phase rise to a peak followed by a decline to a nadir, and a second phase rise to a plateau (Normo-Hyper test); Subject 24, a two phase response showing a rise to a peak followed by a decline for both primary and secondary phases (Hypo-Hyper test); Subject 25, a two phase response showing a primary phase rise to a peak followed by a decline to a nadir, and a secondary plateau phase (Hypo-Hyper test); Subject 22, a response showing an oscillation before reaching a plateau (Normo-Hyper test).

Figure 1 shows examples of the types of transient responses observed, and each of these responses is shown in detail, including all measured variables, in the supplementary information figures. Most of the responses were composed of two phases; only a few could be viewed as a simple rise to a plateau or a rise to a peak followed by a decline. Two-phase responses occurred in 75% of the Hypo-Hyper tests and in 64% of the Normo-Hyper tests. The mean (SD) time to the nadir delineating the two phase responses was 142 (59) s.

The initial response to the step increase in CO_2_ tension was fast in all subjects, with an overall mean (SD) exponential rise time constant of 20.2 (9.6) s. However, in some five individuals the initial responses were oscillatory, especially during the Normo-Hyper CO_2_ steps, as subject 22 in Figure 1 illustrates. The mean (SD) period of this oscillation was 110 (13) s.

We noted qualitative differences in the primary-phase responses between the two CO_2_ step ranges used. The peak MCAv attained before the decline to a nadir was higher for the Hypo-Hyper CO_2_ steps and the subsequent decline was longer. The main differences in the secondary phases were a predominance of slow increases in the Normo-Hyper tests, but plateaus for the Hypo-Hyper tests.

## Discussion

### General Findings

The main finding of this study is that the time course of MCAv responses to a single, sustained, isoxic 10 mmHg step increase in CO_2_ tension, whether from baseline or from 5 mmHg below baseline, is complex. Although the dynamic characteristics of the responses differed between subjects, as well as between the two CO_2_ step ranges within a subject, nevertheless, the majority of subjects at both ranges of CO_2_ step stimuli had MCAv responses that were divisible into two phases delineated by the early decline of the initial response to a visible nadir. These findings have two important implications. First, the cerebrovascular control system can exhibit instability. Second, determinations of cerebrovascular responsiveness to CO_2_ should not assume that measurement at a single time point is sufficient to characterise the response.

The initial response to the step increase in CO_2_ tension was fast in all subjects, with an overall mean (SD) exponential rise time constant of 20.2 (9.6) s; shorter than that of 45 s reported by Poulin et al. [12]. Differences between our measures and those reported by others may reflect the more rapid isoxic change of PaCO_2_ afforded by our new control technology, as well as the greater time resolution (4 s compared to 15 s sampling periods) of our measurements. Indeed the rapid response time in this study is similar to that reported by Cui et al. [23] who also used this technology.

For the initial phase of the responses we noted that the peak MCAv attained above baseline was higher for the Hypo-Hyper CO_2_ tests than the Normo-Hyper tests and the duration of decline was longer. Since the baseline PaCO_2_ is near the midpoint of the sigmoidal-shaped PaCO_2_-CBF response curve [24], a 10 mmHg change in PaCO_2_ about this resting PaCO_2_ value likely places the response on the steepest part of this curve. By contrast, the Normo-Hyper protocol includes the upper flattened part of the response curve so that responses are smaller.

For the secondary phase of the responses we noted that steady responses were mostly associated with the Hypo-Hyper CO_2_ range while responses consisting of a single rise were mostly associated with the Normo-Hyper CO_2_ range. While secondary phase responses consisting of a rise followed by a decline were present for both CO_2_ step ranges, they were in the minority; present in 4 subjects for each CO_2_ stimulus range. For such responses, although the extent of the decline from the peak was not large, it was nevertheless present in these subjects, in agreement with the findings of Ellingsen et al. [25], but not Poulin et al. [12].

Remarkably, in some five individuals the responses were oscillatory, especially during the Normo-Hyper CO_2_ steps, as Subject 22 in Figure 1 illustrates. The mean (SD) period of this oscillation was 110 (13) s, which coincidently is similar to the time constant for the central respiratory chemoreflex response to hypercapnia [26] dictated by the cerebral volume to flow ratio.

### Possible Mechanisms

In seeking possible explanations for our observations we considered two features of the cerebral circulation. First we note that the overall cerebrovascular response to a global increase in CO_2_ tension is complicated by local regulatory factors that control the distribution of cerebral blood flow [27]. They include both neural activity [28], [29] and changes in local perfusion pressure [30], [31] acting within particular vascular regions [32]; the latter observable as an overall autoregulation of cerebral blood flow [33]. With such a fast CO_2_-induced vasodilation, it is possible that a myogenic counter-reaction of the autoregulation mechanism [32], [34] may have been activated that produced the nadir delineating the two phases of the response. However, this explanation for the features of the dynamic responses we observed must remain speculative until further experimental investigation.

An alternative explanation arises from the competition for flow between various cerebrovascular beds, which has been detected in response to system disturbances such as hypoxia [35] as well as hypercapnia [36], [37]. Indeed, recent steady-state studies show that the cerebrovascular reactivity to CO_2_ differs between the territories perfused by vertebral and internal carotid arteries [38], [39]. As van der Zande et al. [6] described, white matter reactivity is about 1/3 of grey matter and is delayed in onset. And the cerebrovascular reactivity to CO_2_ of grey matter is robust and fast, whereas that of white matter is weak and slow [4]. Similarly, we speculate that MCA flow is apportioned between its various branches, which may compete for flow with different magnitudes and response times resulting in the dynamic responses we observed. Again this speculation must await further experimentation.

### Limitations

The patterns of MCAv response we observed could result from similar patterns in the PetCO_2_ stimulus, perfusion pressure, heart rate (reflective of cardiac output), or ventilation. However, as the example figures in the supporting information show, and the stimulus characteristics confirm, isocapnia and isoxia were well maintained throughout the hypercapnic step increases. Nor were the response patterns determined by similar patterns in heart rate, blood pressure or ventilation. Blood pressure was unchanged throughout the testing period for the Hypo-Hyper CO_2_ tests. During the Normo-Hyper CO_2_ tests blood pressure rose slowly over the course of the increased CO_2_ for half of the subjects by a mean (SD) of 9.9 (2.73) mmHg. We suggest that such a small slow increase would have a minimal effect on the observed time course of MCAv.

Neither do we believe that the patterns of MCAv we observed were an artefact of the insonation technique. The mean (SD) insonation depths for the left and right probes were 48 (2.7) and 49.1 (3.5) mm respectively, within the range of a frequently used standard for identification of the M1 [18], [40]. In several subjects both the M1 and M2 could be simultaneously displayed while selecting depths as described by Alexandrov et al. [18]. Although the ACA depth overlaps with the M1 depth territory (50–78 mm), its direction of flow is the opposite of the M1 and therefore easily differentiated using PM-TCD. Based on the depth of insonation and direction of flow we are convinced that we have accurately selected for the M1.

When using velocity measurements as an index of flow within a vessel it is assumed that the vessel diameter does not change. If so, then our observations may have reflected such changes rather than only flow. We do not think that such is the case. In the range of changes in PaCO_2_ used in our studies [10], [21] the changes in MCAv are small, and MCAv has been widely promoted as good a surrogate for CBF as any other method [41]. The MCA, as one of the larger basilar vessels, receives 80% of the internal carotid artery blood flow [42]. Over the range of PCO_2_ in this study it functions as a conductive vessel, as changes in diameter are reported considerably less than 5% under conditions that affect resistance vessels such as hypercapnia [43] and hypocapnia [44]. As the MCA in adults is a rather large vessel (1.5–3 mm in diameter), any such diameter changes would result in less than 4–6% change in MCAv reading for the same flow. Furthermore, in subjects undergoing provocations to alter CBF, there is a strong correlation between resulting changes in MCAv and changes in CBF as measured by “reference standard” techniques such as 133Xe SPECT9 10 and electromagnetic flow probes on the ipsilateral carotid artery [45]. Although historically debated [46], the overwhelming balance of experimental evidence leaves little doubt that under most conditions, changes in TCD velocity signal are directly related to changes in CBF. In any event, TCD is commonly used in studies as a surrogate for CBF and our findings of time related variations in TCD readings in response to a step change in PCO_2_ must be accounted for.

Finally, we emphasise that the responses were only measured once in each subject. As a result we cannot determine if they were repeatable or part of a range of responses that occur in a single individual. Repeatability studies and studies under varied conditions would inform to what extent such responses are characteristic of the cerebral blood flow regulation for an individual.

### Conclusions

We show that the dynamic response to an isoxic step increase in CO_2_ tension measured in MCAv is complex, and differs between subjects and for different stimulus ranges. Thus the control of cerebral blood flow can exhibit instability when stimulated with rapid changes in CO_2_ tension. We suggest that these transient adjustments should be taken into consideration when making measurements of CO_2_ reactivity; a steady state may not be established after rapid changes in CO_2_ for several hundred seconds.

These MCAv response patterns may reflect the dynamic interaction of the CO_2_ vasodilatory stimulus with the pressure autoregulation mechanism, and possibly the dynamic redistribution of blood flow between the various branches of the MCA. However, the physiological mechanisms underlying these dynamic responses must await further investigation.

Our findings illustrate the variety of MCAv responses to an isoxic step increase in CO_2_ tension found in healthy people. The question of whether certain patterns of response are characteristic of an individual must wait upon a reproducibility study.

## Supporting Information

Figure S1 **An example (subject 1) of a single exponential rise response (Exp).** Breath-by-breath values of PetCO_2_ mmHg (squares), PetO_2_ mmHg (circles), and Ve L/min (small triangles), beat-by-beat HR bpm (+) and MAP mmHg (x), and 4-s averages of right MCAv cm/s (open diamonds) and left MCAv cm/s (filled diamonds).
(TIF)

Figure S2 **An example (subject 2) of an exponential rise and fall response (Dual Exp).** Breath-by-breath values of PetCO_2_ mmHg (squares), PetO_2_ mmHg (circles), and Ve L/min (small triangles), beat-by-beat HR bpm (+) and MAP mmHg (x), 4-s averages of right MCAv cm/s (open diamonds) and left MCAv cm/s (filled diamonds).
(TIF)

Figure S3 **An example (subject 12) of a two phase response with a primary phase exponential rise and fall (Dual Exp), and a secondary exponential rise (Exp).** Breath-by-breath values of PetCO_2_ mmHg (squares), PetO_2_ mmHg (circles) and Ve L/min (small triangles), beat-by-beat HR bpm (+) and MAP mmHg (x), 4-s averages of right MCAv cm/s (open diamonds) and left MCAv cm/s (filled diamonds).
(TIF)

Figure S4 **An example (subject 24) of a two phase response with a primary phase exponential rise and fall (Dual Exp), and a secondary exponential rise and fall (Dual Exp).** Breath-by-breath values of PetCO_2_ mmHg (squares), PetO_2_ mmHg (circles) and Ve L/min (small triangles), beat-by-beat HR bpm (+) and MAP mmHg (x), 4-s averages of right MCAv cm/s (filled diamonds) and left MCAv cm/s (open diamonds).
(TIF)

Figure S5 **An example (subject 25) of a two phase response with a primary phase exponential rise and fall (Dual Exp), and a secondary steady mean (Mean).** Breath-by-breath values of PetCO_2_ mmHg (squares), PetO_2_ mmHg (circles) and Ve L/min (small triangles), beat-by-beat HR bpm (+) and MAP mmHg (x), 4-s average right MCAv cm/s (filled diamonds) and left MCAv cm/s (open diamonds).
(TIF)

Figure S6 **An example (subject 22) of an oscillatory response.** Breath-by-breath values of PetCO_2_ mmHg (squares), PetO_2_ mmHg (circles) and Ve L/min (small triangles), beat-by-beat HR bpm (+) and MAP mmHg (x), 4-s average right MCAv cm/s (filled diamonds) and left MCAv cm/s (open diamonds).
(TIF)
